# Chitosan-Stabilized Selenium Nanoparticles Alleviate High-Fat Diet-Induced Non-Alcoholic Fatty Liver Disease (NAFLD) by Modulating the Gut Barrier Function and Microbiota

**DOI:** 10.3390/jfb15080236

**Published:** 2024-08-22

**Authors:** Yuhang Luo, Shujiang Peng, Jintao Cheng, Hongli Yang, Lin Lin, Guiling Yang, Yuanxiang Jin, Qingchi Wang, Zhengshun Wen

**Affiliations:** 1 School of Food and Pharmacy, Zhejiang Ocean University, Zhoushan 316022, China; 2 Xianghu Laboratory, Hangzhou 311231, China; 3 College of Biotechnology and Bioengineering, Zhejiang University of Technology, Hangzhou 310032, China

**Keywords:** low molecular weight chitosan selenium nanoparticles, intestinal barrier function, non-alcoholic fatty liver disease, intestinal microbiota, metabolomics

## Abstract

Low molecular weight chitosan selenium nanoparticles (LCS-SeNPs), a biologically active compound derived from selenium polysaccharides, have demonstrated potential in addressing obesity. However, the mechanism through which LCS-SeNPs alleviate high-fat diet (HFD)-induced non-alcoholic fatty liver disease (NAFLD) remains unclear. Our results elucidated that LCS-SeNPs significantly inhibited fat accumulation and markedly improved the intestinal barrier by increasing mucus secretion from goblet cells. Moreover, LCS-SeNPs reshaped intestinal flora composition by increasing the abundance of mucus-associated microbiota (*Bifidobacterium*, *Akkermansia*, and *Muribaculaceae_unclassified*) and decreasing the abundance of obesity-contributed bacterium (*Anaerotruncus*, *Lachnoclostridium*, and *Proteus*). The modulation of intestinal microbiota by LCS-SeNPs influenced several metabolic pathways, including bile acid secretion, purine metabolites, and tryptophan derivation. Meanwhile, glycocholic acid and tauro-beta-muricholic acid were significantly reduced in the LCS-SeNP group. Our study suggests the crucial role of intestinal microbiota composition and metabolism, providing a new theoretical foundation for utilizing selenium polysaccharides in the intervention of HFD-induced NAFLD.

## 1. Introduction

Non-alcoholic fatty liver disease (NAFLD) induced by a high-fat diet (HFD) is a clinical pathological syndrome characterized by hepatocyte lipid degeneration, which is an important predisposing factor for the development of hepatocellular carcinoma and cirrhosis [1,2,3]. Moreover, the development of NAFLD is accompanied by the disruption of the intestinal epithelial barrier, leading to an increase in intestinal permeability and the translocation of gut bacteria [4,5]. Previous studies have shown that the gut microbiota can impact multiple metabolic pathways, including tryptophan metabolism, primary bile acid biosynthesis, and glycerolipid metabolism, which contribute to reducing inflammation and modulating lipid metabolism in NAFLD patients [6]. However, there is currently no approved drug therapy for NAFLD [7]. Dietary adjustments and exercise regimens represent the effective therapeutic options for NAFLD [8].

Selenium polysaccharides represent a novel functional polysaccharide product formed by the combination of inorganic selenium with polysaccharides [9]. Additionally, multiple studies have shown that selenium polysaccharides exhibit remarkable effects in lowering blood lipids, reducing blood glucose, and modulating immune function [10,11,12]. Moreover, selenium supplementation enhances the growth of beneficial gut microbiota and influences the enrichment of associated metabolic pathways [13]. For example, selenium-enriched kiwi fruit was demonstrated to alleviate hyperlipidemia in HFD mice by significantly increasing the relative abundance of beneficial gut bacteria such as *Parabacteroides, Bacteroides,* and *Allobaculum*, as well as influencing the pyrimidine metabolism and purine metabolism pathways [14].

Chitosan (CS) is a naturally occurring cationic polysaccharide derived from chitin, ranking as the world’s second most abundant biopolymer [15]. Moreover, CS has demonstrated broad biological activity in several fields, including anti-obesity, anti-inflammatory, and immune modulation [16]. Previous studies have shown that low molecular weight CS enhanced intestinal barrier integrity and reduced inflammation in HFD mice by increasing the relative abundance of the beneficial gut bacteria *Akkermansia* and *Gammaproteobacteria* while decreasing the relative abundance of the inflammatory bacteria *Erysipelatoclostridium* and *Alistipes* [17]. In addition, CS also exhibits remarkable drug delivery capabilities attributable to its superior solubility, biocompatibility, biodegradability, and structural modifiability [15,18,19]. For instance, Sen Li et al. developed a targeted nanoparticle coated with *N*-trimethyl chitosan designed to promote sustained release in the intestine [20]. This formulation significantly enhanced the bioavailability and antioxidant activity of vitexin in vivo. Thus, nanoparticles constructed from chitosan for encapsulating bioactive compounds may represent an effective strategy for alleviating NAFLD.

According to our previous research, low molecular weight chitosan selenium nanoparticles (LCS-SeNPs) have demonstrated excellent anti-inflammatory, antioxidant, and gut barrier function-improving effects [21]. To investigate whether LCS-SeNPs alleviate HFD-induced NAFLD, we established the HFD mouse model. Herein, we determined the efficacy of LCS-SeNPs in alleviating HFD-induced intestinal barrier dysfunction. Next, we explored the relationship between key microbial species altered by LCS-SeNPs and colon metabolites in relation to alleviating NAFLD. Ultimately, the findings of this study may offer a novel insight into prevention and treatment strategies for NAFLD.

## 2. Materials and Methods

### 2.1. Materials

Low molecular weight chitosan (LCS) with a molecular weight (MW) of 3000 Da and a deacetylation degree (DD) of 90% (DD 90%) was purchased from Golden-Shell Pharmaceutical Co., Ltd. (Zhejiang, China). The same LCS was used for the synthesis of low molecular weight chitosan selenium nanoparticles (LCS-SeNPs). Thermo Fisher Scientific (Waltham, MA, USA) furnished methanol, formic acid, and acetonitrile at High-Performance Liquid Chromatography (HPLC) grade. Sodium selenites were purchased from Sigma-Aldrich (St. Louis, MO, USA). Ethanol absolute and glacial acetic acid were obtained from Shanghai Macklin Biochemical Co., Ltd. (Shanghai, China). The remaining chemicals utilized were of analytical grade. 

### 2.2. Preparation of Low Molecular Weight Chitosan Selenium Nanoparticles

The LCS-SeNPs used in this study were synthesized using LCS from our previous work [21]. In brief, 1 g chitosan was dissolved in 49 mL distilled water, followed by the addition of 1 mL acetic acid and 10 mg sodium selenite. The mixture was heated at 60 °C for 4 h, filtered through a 0.2 μm membrane after standing overnight, and confirmed sodium selenite-free via ascorbic acid testing. LCS-SeNPs were obtained through vacuum freeze-drying.

### 2.3. Animal Experiments

The Guidelines for the Care and Use of Laboratory Animals were followed in this study and approved by the Animal Ethics Committee of Zhejiang Ocean University (authorization number: 2022025). All mice were housed according to the requirements of the committee in the standard-specific pathogen-free level animal facility of Zhejiang Ocean University. Healthy male C57BL/6J mice (5-week-old, 18 ± 2 g) purchased from Charles River Laboratory (Jiaxing, China) were raised in a standardized environment (12 h light/12 h dark cycle, temperature 23 ± 1 °C, relative humidity 55 ± 5%). Adhering to the principle of ad libitum feeding, four mice were housed in each cage (each cage had two compartments, and each compartment contained two mice).

**Figure 1:**
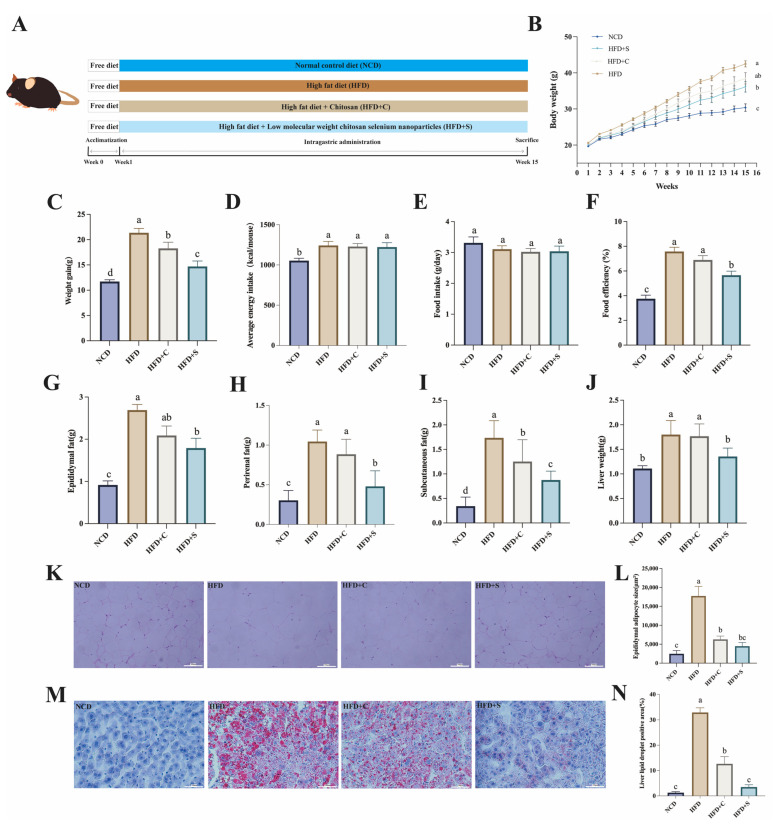
LCS-SeNP intervention in HFD mice effectively curtailed fat accumulation. (**A**) Overview of the entire experimental process; (**B**) Trend of body weight in mice after a 14-week dietary intervention; (**C**) Average body weight gain of mice in each group throughout the entire experimental duration; (**D**) The average energy intake of each mouse at the end of the experimental period; (**E**) Daily food intake; (**F**) Food efficiency = body weight gain/food intake; Weight measurements for (**G**) epididymal lipid, (**H**) perirenal lipid, (**I**) subcutaneous lipid, and (**J**) liver; (**K**) Sections of epididymal adipose tissue were stained with H&E (proportional scale: 100 μm); (**L**) The epididymal adipocyte size (μm^2^); (**M**) Oil Red O staining of the liver section (proportional scale: 100 μm); (**N**) the liver lipid droplet positive area (%). Data are presented as the mean ± SEM (n = 6). The distinct letters above the bar chart indicated significant differences (*p* < 0.05). LCS-SeNPs, low molecular weight chitosan selenium nanoparticles; HFD, high-fat diet; H&E, Hematoxylin and Eosin; SEM, standard error of the mean.

After one week of acclimatization, all mice were randomly assigned to four groups (n = 6) for the dietary intervention: (1) normal chow diet (NCD) group (D12450J, 4.3% fat); (2) high-fat diet (HFD) group (D12492, 35% fat); (3) high-fat diet with low molecular weight chitosan (HFD+C) (200 mg/kg/day); (4) high-fat diet with low molecular weight chitosan selenium nanoparticles (HFD+S) (200 mg/kg/day). Administration was conducted by intragastric gavage in this study. The entire experimental process is delineated in Figure 1A.

The experimental duration for each group spanned 14 weeks, with weekly recordings of body weight gain and food intake data. After a 12 h fasting period, mouse serum samples were obtained by retroorbital bleeding, and the resultant serum was stored at −80 °C. All mice were sacrificed by cervical dislocation following anesthesia with ether. We carefully dissected and removed the subcutaneous fat, perirenal fat, liver fat, and epididymal fat tissues from each group of mice under a microscope and precisely measured the weight of each tissue sample. The collected liver, epididymal fat, and colon tissues were partially fixed in 4% paraformaldehyde for subsequent histopathological analysis. The remaining portions, as well as the colonic contents, were rapidly frozen in liquid nitrogen and stored at −80 °C for further analysis. 

### 2.4. Serum Analysis

Blood samples were obtained by retroorbital bleeding, and serum was collected after centrifugation (1000 g, 5 min). Serum samples from each group of mice were used to assess the relevant indicators, including triglyceride (TG) (E-BC-K261-M), aspartate aminotransferase (AST) (E-BC-K236-M), total cholesterol (TC) (E-BC-K109-M), and alanine aminotransferase (ALT) (E-BC-K235-M) using commercial kits purchased from Elabscience Biotechnology Co., Ltd. (Wuhan, China). The levels of lipopolysaccharide (LPS) (CSB-E13066m), diamine oxidase (DAO) (CSB-E10090m), interleukin-6 (IL-6) (CSB-E04639m), and tumor necrosis factor (TNF-*α*) (CSB-E04741m) were measured in the serum using commercial kits purchased from CUSABIO BIOTECH CO., Ltd. (Wuhan, China).

### 2.5. Histological Analysis

The epididymal fat was subjected to Hematoxylin and Eosin (H&E) staining to observe tissue morphology. Subsequently, histological evaluation of the liver involved the application of both Oil Red O staining and H&E staining techniques. The colon tissue was stained with H&E staining and Alcian Blue-Periodic Acid-Schiff (AB-PAS) staining. Sections were observed under a microscope (Carl Zeiss AG, Oberkochen, Germany).

### 2.6. NAFLD Activity Score

The NAFLD Activity Score is a scoring system used to assess the histological severity of liver tissue in patients with NAFLD. It evaluates three key histological features in liver biopsy samples: hepatocellular steatosis, inflammation of hepatic lobules, and ballooning of hepatocytes, and provides a total score based on the combined evaluation of these three factors [22].

### 2.7. Transmission Electron Microscopy

Colon samples designated for Transmission Electron Microscopy (TEM) were fixed in a solution of 2.5% glutaraldehyde and 1% osmic acid at 4 °C overnight. After three 15 min wash cycles in 0.1 M PBS (pH = 7.0), the samples underwent ethanol and acetone dehydration steps. Osmotic embedding was performed at 37 °C using a mixture of acetone and EMBed 812 (Servicebio, Wuhan, China), followed by polymerization. Ultrathin sections (60–80 nm) were stained with uranyl acetate and lead citrate and observed using the HT7800 TEM (Hitachi, Tokyo, Japan).

### 2.8. RNA Isolation and Real-Time PCR

Total RNA from the liver and colon tissues was extracted using the RNAeasy™ animal RNA extraction kit (Solarbio Science & Technology Co., Ltd., Beijing, China) according to the manufacturer’s instructions. The resulting RNA solution was immediately stored in a refrigerator at −80 °C. Fluorescent quantitative PCR reactions were conducted using the TB Green^®^ Premix Ex Taq™ kit (Takara Biomedical Technology Co., Ltd., Shiga, Japan) following the manufacturer’s instructions. The specific primers used and their sequences are listed in Table S1. The reaction was set up in a 20 μL system, and the volumes of each reaction component were as indicated in Table S2. Quantifying the mRNA expression levels of the target genes using the 2^-ΔΔCt^ method involved employing β-actin as the reference gene.

### 2.9. DNA Extraction and Sequencing

Total fecal microbial DNA was obtained through the Fecal Genome DNA extraction kit (BioTeke, Wuxi, China) according to the manufacturer’s instruction manual. DNA was quantified by Qubit (Invitrogen, Carlsbad, CA, USA). We utilized the universal primer 341F/805R; the specific sequences are shown in Table S3. The PCR products were subjected to purification using AMPure XT beads (Beckman Coulter Genomics, Danvers, MA, USA) and quantification with Qubit. The quality of the PCR products was assessed using Agilent 2100 Bioanalyzer (Santa Clara, CA, USA) and Illumina library quantitative kits (Kapa Biosciences, Woburn, MA, USA). LC-Bio Technology Co., Ltd. (Hangzhou, China) uniformly pooled qualified PCR products and conducted sequencing on the Illumina NovaSeq 6000 (PE250) platform.

### 2.10. Microbiota Data Analysis

Sequencing of the samples was conducted on the Illumina NovaSeq platform following the guidelines provided by LC-Bio Technologies Co., Ltd. Original reads were filtered using fqtrim (version 0.94) under specific filtering conditions to obtain high-quality clean labels. Chimeric sequences were identified using Vsearch software (version 2.3.4). Alpha diversity and beta abundance were calculated using QIIME2 (version 2023.7) and visualized using the R package (version 4.0.3). To evaluate the similarities among microbial communities across different samples, we conducted a principal coordinate analysis (PCoA) based on Bray–Curtis dissimilarity, utilizing the Vegan v2.5–3 package. A volcano plot was generated by plotting log2 fold change (FC, FC > 2 or <0.5) against –log10 *p*-value (*p* > 0.05) using R software (v2.15.3). The generation of all additional figures was accomplished utilizing the R package.

### 2.11. Untargeted Metabolite Analysis of Intestinal Contents

The intestinal contents of the four groups of mice were subjected to metabolomic analysis. Metabolite profiling was conducted using LC-MS/MS-based metabolomics methodology by LC-Bio Technologies Co., Ltd. Initially, chromatographic separation of all samples was accomplished utilizing a Vanquish Flex UHPLC system manufactured by Thermo Fisher Scientific. Reversed-phase separation was performed on an ACQUITY UPLC T3 column (100 mm × 2.1 mm, 1.8 µm, Waters, Milford, MA, USA).

Peak intensity data derived from the mass spectra were normalized and processed using the R package, and exact molecular mass data (*m/z*) of samples were aligned with the Kyoto Encyclopedia of Genes and Genomes (KEGG) for metabolite annotation. Establishing criteria to discern distinctions between the two sets of metabolites involved parameters such as the variable importance in projection (VIP > 1), fold change (FC > 2 or <0.5), and Student’s *t*-test (*p* < 0.05). Supervised Partial Least Squares Discriminant Analysis (PLS-DA) was performed with the R package to distinguish different variables between groups. 

### 2.12. Statistical Analysis

GraphPad Prism 9 (GraphPad Software, San Diego, CA, USA) was used for statistical testing, and one-way analysis of variance and the Tukey test were used for significant differences analysis. The Student’s *t*-test was employed to assess differences between the two groups. All the findings were presented in terms of the mean values accompanied by the standard error of the mean (SEM). The threshold for establishing statistical significance was defined as a *p* < 0.05. A heatmap was generated using Spearman’s correlation analysis to calculate the relationship between different metabolites and microbial community diversity.

## 3. Results

### 3.1. LCS-SeNPs Inhibited Body Weight Gain and Alleviated Dyslipidemia

To investigate the effect of LCS-SeNPs on inhibiting lipid accumulation in mice fed HFD, we monitored the body weights of mice in each group throughout the experimental period. The results indicated the LCS-SeNP group accumulated significantly less body weight than the HFD group throughout the experimental period (Figure 1B,C). Following 14 weeks of dietary intervention, notable distinctions in energy consumption were observed between the NCD group and the treatment groups, whereas no significant alterations were found among the groups subjected to HFD (Figure 1D). As illustrated in Figure 1E, no significant differences in food intake were observed between the groups, suggesting that the reduction in weight gain observed in the HFD+S group was not attributable to variations in food consumption. Additionally, food efficiency in the HFD+S group was significantly lower compared with the HFD group (Figure 1F). In contrast, although the HFD+C group exhibited lower food efficiency than the HFD group, this difference was not statistically significant. These results indicated that the LCS-SeNP group did not reduce the weight gain of HFD by reducing the appetite of mice. Additionally, the LCS-SeNPs-treated mice demonstrated significantly reduced weights in epididymal, perirenal, subcutaneous fat, and liver, and the liver weight in the LCS-SeNP group displayed no statistically significant disparity in comparison to the NCD group **(**Figure 1G–J). Moreover, the results of H&E staining of epididymal adipose tissue and Oil Red O staining of the liver demonstrated the anti-obesity effect of LCS-SeNPs in HFD mice (Figure 1K–N).

### 3.2. LCS-SeNPs Attenuated Hepatic Steatosis in HFD-Fed Mice through Modulation of Lipid Metabolism

**Figure 2:**
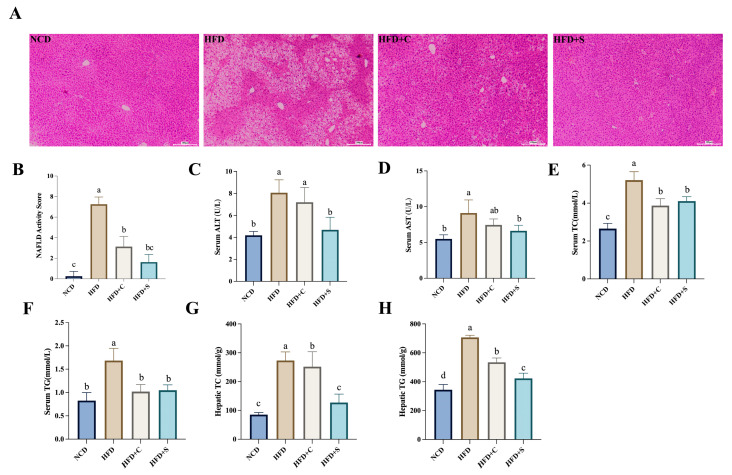
Variations in liver and blood lipid parameters among mice in different treatment groups. (**A**) Sections of liver tissue were stained with H&E (proportional scale: 100 μm); (**B**) NAFLD activity score; (**C**) serum ALT; (**D**) serum AST; (**E**) serum TC; (**F**) serum TG; (**G**) Hepatic TC; (**H**) Hepatic TG. Data are presented as the mean ± SEM (n = 6). The distinct letters above the bar chart indicated significant differences (*p* < 0.05). H&E, Hematoxylin and Eosin; NAFLD, nonalcoholic fatty liver disease; ALT, alanine aminotransferase; AST, aspartate aminotransferase; TC, total cholesterol; TG, triglyceride; HFD, high-fat diet; SEM, standard error of the mean.

After evaluating the weight-reducing effects of LCS-SeNPs, we proceeded to investigate the effect of LCS-SeNPs on the liver of HFD-fed mice. According to the H&E staining, observations revealed that the LCS-SeNP intervention alleviated hepatic cell lipid degeneration, disorderly arrangement, ballooning degeneration, and inflammatory cell infiltration induced by an HFD (Figure 2A). Furthermore, the LCS-SeNP group exhibited significantly reduced NAFLD activity scores compared with the HFD group, with no statistically notable divergence observed between the LCS-SeNPs and NCD groups (Figure 2B). NAFLD patients are typically identified by asymptomatic elevation of liver enzymes, with the most common being ALT and AST [23,24]. Serum indicators showed that ALT and AST levels in the serum of HFD mice were significantly decreased after the LCS-SeNP intervention (Figure 2C,D), but no statistically significant differences were observed when compared with the NCD group. Furthermore, LCS-SeNPs significantly improved TC and TG levels, indicating their potent efficacy in ameliorating dyslipidemia in HFD-fed mice (Figure 2E–H).

### 3.3. LCS-SeNPs Ameliorated Intestinal Mucosal Barrier and Inflammatory Responses in HFD-Fed Mice

**Figure 3:**
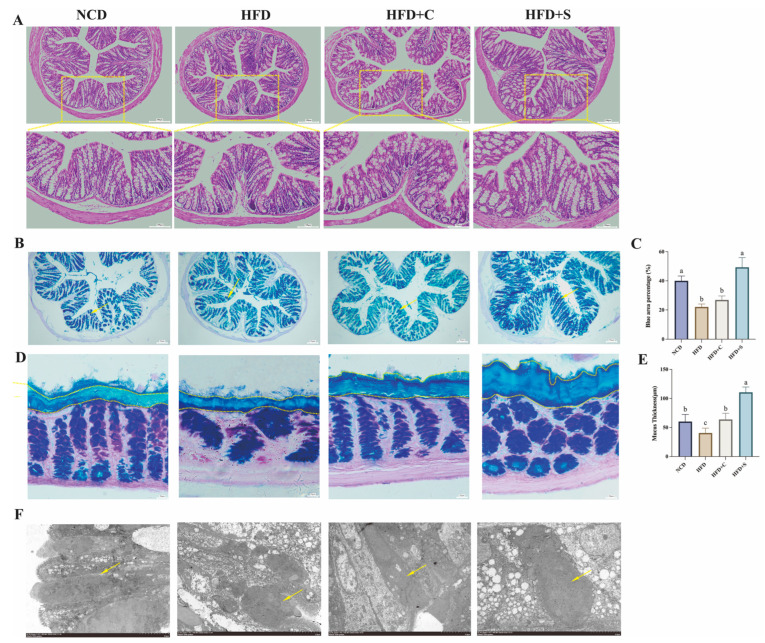
Influences of LCS-SeNPs on colon structure and secretion in mice subjected to an HFD. (**A**) Sections of colonic segments were stained with H&E (proportional scale: 100 μm); (**B**) AB-PAS staining of colon tissue without fecal content (proportional scale: 50 μm), the arrows in (**B**) indicate the stained goblet cells; (**C**) Blue area proportion (%); (**D**) Colonic mucus was stained with AB-PAS (proportional scale: 20 μm); (**E**) Mucus thickness (μm); (**F**) The representative ultrastructure of colonic goblet cells in each group (magnified 2.5 k times), the arrows in (**F**) indicate mucin granules. Data are presented as the mean ± SEM (n = 6). The distinct letters above the bar chart indicated significant differences (*p* < 0.05). LCS-SeNPs, low molecular weight chitosan selenium nanoparticles; HFD, high-fat diet; H&E, Hematoxylin and Eosin; AB-PAS, alcian blue-periodic acid-schiff; SEM, standard error of the mean.

**Figure 4:**
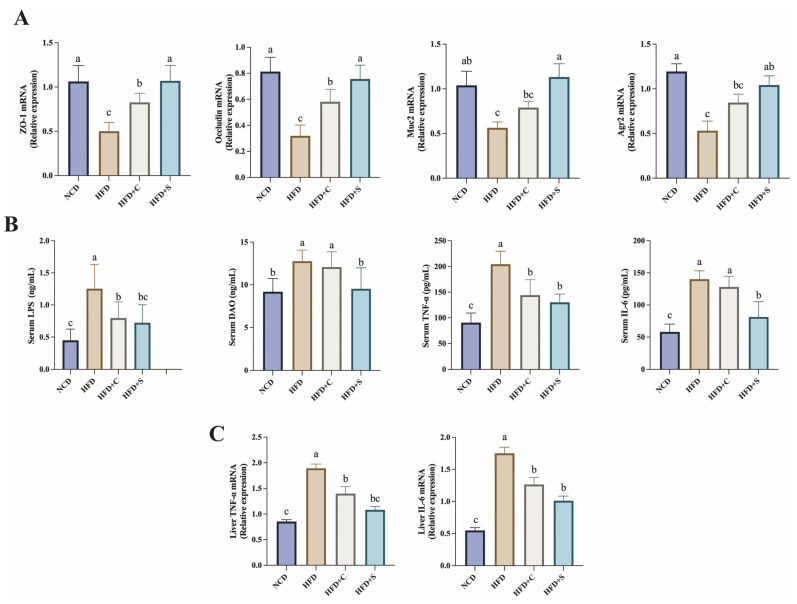
Treatment with LCS-SeNPs enhances intestinal barrier function in HFD-fed mice. (**A**) The relative mRNA expression levels of *ZO-1*, *occludin*, *Muc2,* and *Agr2* in colon tissue; (**B**) Serum indexes of intestinal mechanical barrier integrity and inflammatory factors include LPS, DAO, TNF-*α*, and IL-6 in HFD-fed mice; (**C**) The relative mRNA expression levels of TNF-*α* and IL-6 in liver. Data are presented as the mean ± SEM (n = 6). The distinct letters above the bar chart indicated significant differences (*p* < 0.05). LCS-SeNPs, low molecular weight chitosan selenium nanoparticles; HFD, high-fat diet; *ZO-1*, Zonula occludens-1; *Muc2*, mucin 2; *Agr2*, anterior gradient 2; LPS, lipopolysaccharide; DAO, diamine oxidase; TNF-α, tumor necrosis factor; IL-6, interleukin-6; SEM, standard error of the mean.

Long-term HFD induces sustained low-grade inflammation and leads to the development of NAFLD [25]. In our study, results of colonic tissue staining with H&E and AB-PAS revealed that LCS-SeNPs improved colonic mucosal epithelial damage induced by HFD, significantly restored the number of goblet cells, and increased intestinal mucus thickness (Figure 3A–E). Moreover, TEM also revealed a significant increase in mucin granules within goblet cells of the colon (shown by the arrow) in mice treated with LCS-SeNPs (Figure 3F), corroborating the findings of AB-PAS staining. To delve deeper into the mechanisms underlying LCS-SeNPs-mediated improvement in intestinal barrier permeability, the relative mRNA expression levels of zonula occludens-1 (*ZO-1*)*, occludin,* mucin 2 (*Muc2*), and anterior gradient 2 (*Agr2*) in the mouse colon were evaluated by real-time PCR (Figure 4A). The findings revealed that LCS-SeNPs significantly elevated the relative mRNA expression levels of *ZO-1*, *occludin*, *Muc2*, and *Agr2* in the colon compared with the HFD group. Disruption of the intestinal barrier allows inflammatory factors to invade the bloodstream [26,27,28]. Hence, we monitored the levels of relevant inflammatory factors in the serum of each group. The results indicated that the levels of LPS, DAO, TNF-*α*, and IL-6 were significantly higher in the HFD group compared with the NCD group (Figure 4B). In contrast, LCS-SeNPs significantly reduced these levels. Moreover, we found that the relative mRNA expression levels of TNF-*α* and IL-6 in the liver were significantly increased in the HFD group, while the LCS-SeNPs significantly decreased these two inflammatory cytokines. These findings indicated that the LCS-SeNP intervention mitigated the intestinal barrier dysfunction induced by an HFD in mice.

### 3.4. Modulatory Effects of LCS-SeNPs on Intestinal Microbiota Composition in HFD Mice

**Figure 5:**
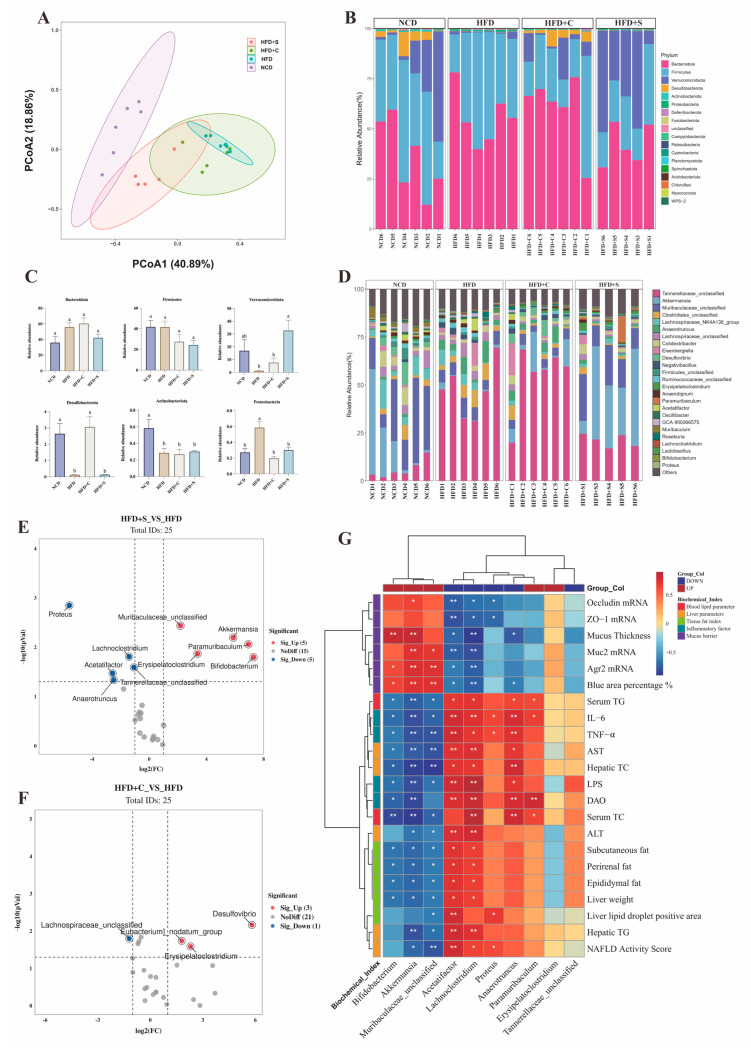
Effect of LCS−SeNPs on the gut flora in HFD−induced obese mice. (**A**) Graph of PCoA scores based on Bray−Curtis; (**B**) Phylum−level configuration of the gut flora; (**C**) The top six bacteria with the highest relative abundance at the phylum level; (**D**) Genus−level configuration of the intestinal flora; Volcano plot analysis based on the gut flora at the genus level (**E**) between HFD+S and HFD groups and (**F**) between HFD+C and HFD groups; (**G**) The association between ten key bacteria and obesity parameters, as well as intestinal barrier parameters. Data are presented as the mean ± SEM (n = 5−6). The distinct letters above the bar chart indicated significant differences (*p* < 0.05). The * symbol in the square highlights the significant correlation between the two (*: *p* < 0.05, **: *p* < 0.01). LCS−SeNPs, low molecular weight chitosan selenium nanoparticles; HFD, high−fat diet; PCoA, principal coordinate analysis; HFD+S, high−fat diet with low molecular weight chitosan selenium nanoparticles; HFD+C, high−fat diet with low molecular weight chitosan; SEM, standard error of the mean.

To further elucidate whether the improvement in body weight and changes in lipid metabolism-related indicators following the intake of LCS-SeNPs in HFD mice are associated with the influence on gut microbiota composition, we performed high-throughput 16S rRNA sequencing of colonic contents. The Chao1 and Shannon rarefaction curves illustrated that the sequencing results adequately capture the diversity present in the current sample (Figure S1A,B). The assessment of bacterial community abundance and diversity involved relative abundance analysis, examination of 6 alpha diversity indices, a Venn diagram, and Principal coordinate analysis (PCoA) (Figure S1C,D and Figure 5A). Samples from the HFD group exhibited a greater distance along the horizontal axis compared with the NCD and LCS-SeNP groups, demonstrating a substantial modification in the structural arrangement of gut microbiota initiated by the HFD. In addition, the community structure histogram showed significant changes in the gut microbiota structure of the HFD+S group at the phylum and genus levels. Specifically, at the taxonomic rank of the phylum, *Firmicutes* and *Bacteroidota* emerged as two predominant phyla in particular. Moreover, the LCS-SeNP group showed an increased abundance of *Verrucomicrobia* compared with the HFD group (Figure 5B,C). At the taxonomic rank of the genus, we observed significant increases in the relative abundances of *Tannerellaceae_unclassified*, *Clostridiales_unclassified*, and *Anaerotruncus* in the HFD group. However, the relative abundances of *Muribaculaceae_unclassified*, *Akkermansia*, and *Paramuribaculum* significantly increased in the HFD+S group, while *Desulfovibrio*, *Muribaculum*, *Acetatifactor*, and *Proteus* showed significant decreases. Additionally, the low molecular weight chitosan (LCS)-fed mice exhibited significant elevations in the relative abundances of *Tannerellaceae_unclassified* and *Eisenbergiella* compared with the HFD group (Figure 5D). Subsequently, differential analysis was separately conducted on the top 25 dominant bacterial genera within the HFD+S and HFD+C groups by setting volcano plot thresholds at fold change > 2 or <0.5 and *p* < 0.05. We observed significant upregulation of five bacterial genera (*Akkermansia, Paramuribaculum*, *Bifidobacterium*, *Erysipelatoclostridium*, and *Muribaculaceae_unclassified*), and significant downregulation of five bacterial genera (*Lachnoclostridium*, *Roseburia*, *Anaerotruncus, Acetatifactor*, and *Proteus*) in the LCS-SeNPs-treated group compared with the HFD group (Figure 5E and Figure S2, Table S4). Meanwhile, *Desulfovibrio, Eubacterium_nodatum_group*, and *Erysipelatoclostridium* exhibited higher relative abundance in the HFD+C group, and *Lachnospiraceae_unclassified* appeared with lower abundance (Figure 5F and Table S5). The results indicate that both LCS and LCS-SeNPs can alter the gut microbiota of obese mice. However, LCS-SeNPs induce more pronounced changes in the gut microbiota. Subsequently, we investigated the relationship between ten key bacteria in the LCS-SeNP group and obesity-related parameters and intestinal barrier parameters by Spearman correlation analysis. The results indicated that *Akkermansia*, *Muribaculaceae_unclassified*, and *Bifidobacterium* exhibited a significant negative correlation with visceral fat weight and a significant positive correlation with mucus barrier indicators *Agr2* mRNA, *Muc2* mRNA, *occludin* mRNA, and mucus thickness. Conversely, *Anaerotruncus*, *Lachnoclostridium*, and *Proteus* showed a positive correlation with visceral fat weight and a negative correlation with mucus barrier indicators. Collectively, these findings confirmed our hypothesis that LCS-SeNPs can alleviate obesity and improve intestinal barrier function by modulating the gut microbiota in HFD mice. 

### 3.5. The Impacts of LCS-SeNPs on Intestinal Metabolomics in HFD Mice

**Figure 6:**
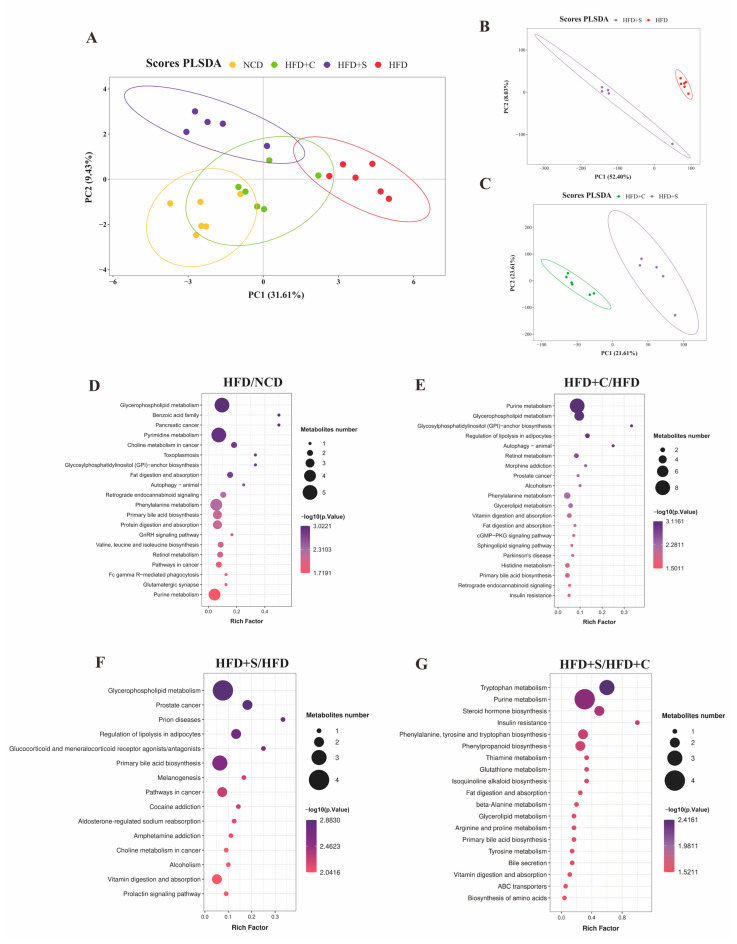
Untargeted metabolomics unveiled alterations in fecal metabolites within the colon of mice subjected to an HFD following LCS−SeNP treatment. (**A**) Analysis of metabolites in NCD, HFD, HFD+C, and HFD+S groups using PLSDA; PLSDA scores of HFD versus LCS−SeNPs (**B**) and LCS versus LCS−SeNPs (**C**); (**D**−**G**) Enrichment analysis of KEGG pathways was performed on the different metabolites between the two groups. Thresholds were applied for differentially abundant metabolites of the KEGG bubble plot (fold change > 2 or <0.5, *p* < 0.05, and VIP value > 1). (n = 5−6). HFD, high−fat diet; LCS−SeNPs, low molecular weight chitosan selenium nanoparticles; NCD, normal chow diet; HFD+C, high−fat diet with low molecular weight chitosan; HFD+S, high−fat diet with low molecular weight chitosan selenium nanoparticles; PLSDA, partial least squares discriminant analysis; LCS, low molecular weight chitosan; KEGG, Kyoto Encyclopedia of Genes and Genomes; SEM, standard error of the mean.

Symbiotic microbial metabolites are considered crucial mediators in host-microbe interactions, with dietary nutrients exerting a significant influence on their regulation [29]. Therefore, to determine whether the intake of LCS-SeNPs can alter the fecal metabolite profile in HFD-induced obese mice, we conducted untargeted metabolomics analysis on the colonic contents of the four groups. The PLSDA results showed the performance of the model without overfitting and revealed distinct separation of fecal metabolic profiles among different groups (Figure 6A–C and Figure S3A,B). A comprehensive analysis identified 124 differential metabolites in the LCS-SeNPs versus HFD group, comprising 81 up-regulated and 43 down-regulated metabolites (Figure S3C). Then, there is a cumulative sum of 101 differential metabolites screened, with 60 exhibiting up-regulation and 41 displaying down-regulation in the LCS-SeNPs versus LCS group (Figure S3D). An enrichment analysis of the Kyoto Encyclopedia of Genes and Genomes (KEGG) pathways for differentially expressed metabolites revealed pathways that were significantly enriched, facilitating the identification of biologically regulated pathways undergoing significant alterations. The criteria for identifying metabolites are set as VIP value > 1 and *p* < 0.05. The KEGG pathway enrichment analysis between the NCD and the HFD groups revealed significant alterations in 20 specific pathways (Figure 6D). These pathways included glycerophospholipid metabolism, pyrimidine metabolism, primary bile acid biosynthesis, and several other pivotal pathways. Furthermore, purine metabolism and glycerophospholipid metabolism represent the top two significantly enriched pathways between the HFD+C and the HFD group (Figure 6E). The divergent metabolites identified between the HFD and LCS-SeNP groups demonstrated notable enrichment in KEGG pathways, such as primary bile acid biosynthesis, glycerophospholipid metabolism, and steroid hormone biosynthesis (Figure 6F). The steroid hormone biosynthesis, tryptophan metabolism, and purine metabolism were the predominant differential metabolites observed between the LCS and LCS-SeNP groups (Figure 6G). Notably, primary bile acid metabolism was one of the most significantly altered pathways in the LCS-SeNP group; this discovery aligned with the findings of a previous study that involved a high-fat diet [29]. Subsequently, we conducted an analysis of significantly different metabolites between the HFD+S and HFD groups (*p* < 0.05). Subsequently, we identified 17 significantly different metabolites closely related to obesity metabolism from the pathways enriched in the KEGG database, which are listed in Table 1. Glycocholic acid, taurine, taurodeoxycholic acid, and tauro-beta-muricholic acid exhibited marked down-regulation in mice subjected to the LCS-SeNP intervention within the context of HFD (Table 1), while 7-Alpha-hydroxy-4-cholesten-3-one was significantly up-regulated. These metabolites were all involved in primary bile acid biosynthesis (Table 1). Moreover, as shown in Figure S4, the fold change value bar chart showed that there were 13 significant metabolites between LCS-SeNPs and LCS. The most enriched metabolites were found in the purine metabolism pathway (deoxyinosine, deoxyguanosine, adenosine, xanthine), and the content of deoxyguanosine was the highest among them, which was consistent with previous research [14]. Followed by tryptophan metabolism (kynurenic acid, indole, 4-(2-aminophenyl)-2,4-dioxobutanoic acid).

### 3.6. Correlation between Gut Microbiota and Fecal Metabolites Following LCS-SeNP Treatment

**Figure 7:**
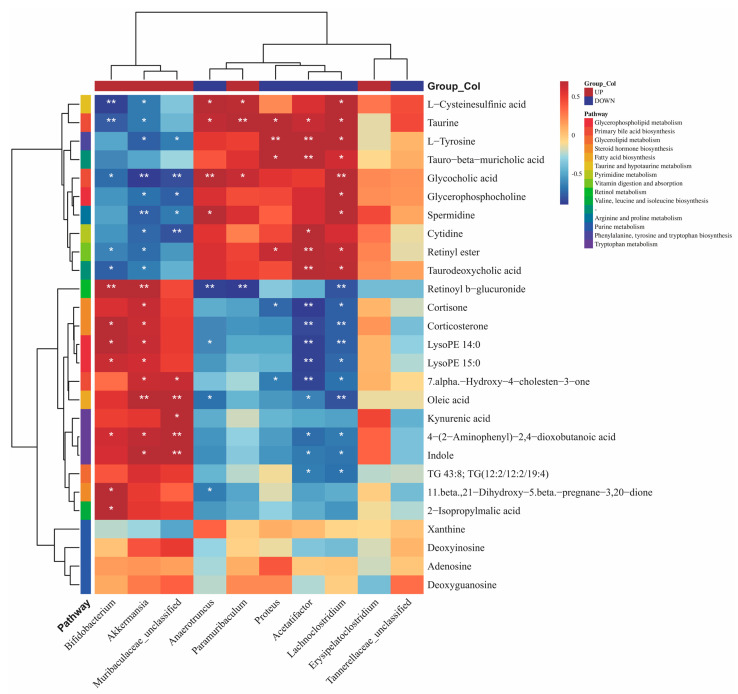
Spearman’s correlation analysis of ten key gut microbiota with different fecal metabolites. Red signifies a positive correlation, while blue indicates a negative correlation, with the depth of the color reflecting the strength of the correlation. The * symbol in the square highlights the significant correlation between the two (*: *p* < 0.05, **: *p* < 0.01).

To further explore the potential relationships between significantly altered metabolites and changes in the gut microbiota, we conducted Spearman’s correlation analysis between the ten key bacterial genera significantly influenced by LCS-SeNPs and the metabolites listed in Table 1. As illustrated in Figure 7, *Akkermansia* exhibited distinct correlations within the pathways of primary bile acid metabolism, glycerophospholipid metabolism, and tryptophan metabolism. For instance, it showed a negative correlation with lipid synthesis-related metabolites such as glycolic acid, tauro-β-muricholic acid, taurodeoxycholic acid, and glycerophosphocholine, while demonstrating a positive correlation with gut homeostasis-maintaining metabolites such as kynurenic acid, indole, and 4-(2-aminophenyl)-2,4-dioxobutanoic acid. *Akkermansia* has been demonstrated to alleviate metabolic disorders induced by an HFD in mice [30,31]. Moreover, given its role in enhancing intestinal barrier function, *Akkermansia* may exert therapeutic effects in preventing metabolic or inflammatory diseases such as obesity, type 1 diabetes, and alcoholic steatohepatitis [32]. *Akkermansia* has been demonstrated as a next-generation probiotic with potential therapeutic effects for treating NAFLD [32]. Furthermore, *Muribaculaceae_unclassified* showed a positive correlation with kynurenic acid, indole, 4-(2-aminophenyl)-2,4-dioxobutanoic acid, and 7-alpha-hydroxy-4-cholesten-3-one. Previous studies have shown that *Muribaculaceae_unclassified* was associated with bile acids and tryptophan metabolites [33]. *Muribaculaceae_unclassified* also plays a key role in improving intestinal structure as well as modulating inflammatory responses [34,35]. In our study, we also found that the genera *Bifidobacterium* and *Lachnoclostridium* were noteworthy contributors between the HFD and LCS-SeNP groups, significantly enriching unique metabolites. A preceding investigation documented that *Bifidobacterium* alleviated HFD-induced NAFLD by modulating the levels of tryptophan metabolites and bile acids [36]. In another investigation, a substantial positive correlation was observed between *Lachnoclostridium* and glycocholic acid, indicating that *Lachnoclostridium* may regulate lipid metabolism by affecting bile acid metabolism to alleviate NAFLD [37]. 

## 4. Discussion

**Figure 8:**
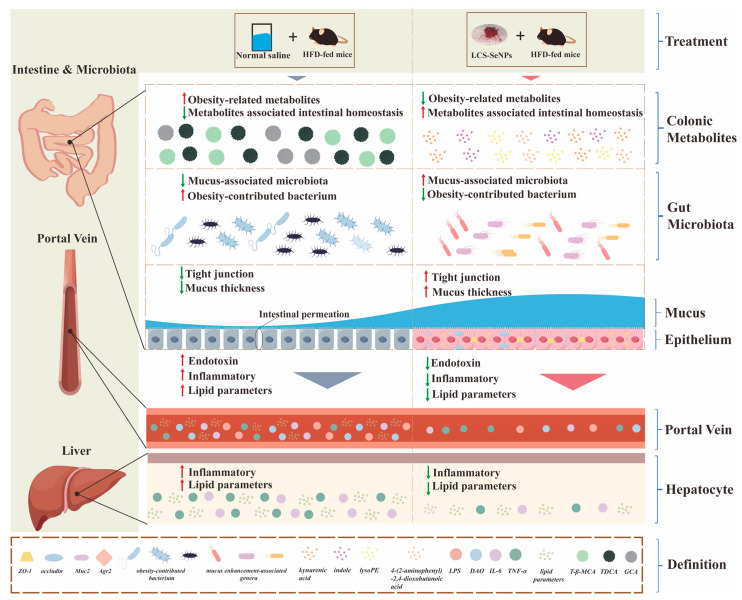
Potential pathways through which LCS-SeNPs alleviate NAFLD and associated metabolic disorders in HFD-fed mice.

In our study (Figure 8), results indicated that the LCS-SeNPs exhibited significant anti-obesity effects after intervening in HFD mice. Herein, we found that LCS-SeNPs significantly reduced body weight in HFD-fed mice and significantly decreased blood lipid levels compared with the HFD group. Additionally, LCS-SeNPs significantly improved intestinal mucus barrier and tight junction in HFD mice, markedly increasing the mRNA expression levels of *ZO1*, *occludin*, *Muc2*, and *Agr2*. The 16S rDNA sequencing analysis showed that LCS-SeNPs significantly increased the abundance of mucus-enhancing genera *Bifidobacterium*, *Akkermansia*, and *Muribaculaceae_unclassified* while decreasing the abundance of obesity-contributing genera *Anaerotruncus*, *Lachnoclostridium*, and *Proteus*. Moreover, metabolomics analysis indicated that LCS-SeNPs significantly upregulated intestinal barrier-supporting metabolites kynurenic acid and indole while decreasing levels of obesity-inducing metabolites, including glycocholic acid and taurodeoxycholic acid. These results suggested that LCS-SeNPs, as a form of selenium polysaccharide, demonstrate excellent efficacy in alleviating NAFLD.

Selenium polysaccharides, as a novel type of functional polysaccharide, not only facilitate absorption and utilization by the human body but also exhibit unique biological activities such as antioxidant, hypoglycemic, and anti-obesity effects [9]. A previous study found that *Ziyang* selenium-enriched green tea polysaccharide significantly increased the relative abundance of probiotic bacteria *Lactobacillus*, *Akkermansia*, and *Bacteroides*, thereby alleviating HFD-induced hepatic steatosis, which was consistent with the results we observed in the LCS-SeNP group where liver tissue degeneration was alleviated (Figure 1M and Figure 2A) [38]. Furthermore, resveratrol-loaded selenium/chitosan nano-flowers (Res@SeNPs@Res-CS-NPs) were demonstrated to reduce LPS levels in HFD mice [39]. Additionally, Res@SeNPs@Res-CS-NPs inhibited obesity and restored intestinal homeostasis by lowering the relative levels of gut microbiota associated with inflammation and lipid deposition [39]. The low molecular weight chitosan selenium nanoparticles (LCS-SeNPs) we prepared demonstrated excellent anti-obesity effects in obese mice. Our results indicated that compared with the model group, LCS-SeNPs significantly reduced the weights of epididymal fat, perirenal fat, subcutaneous fat, and liver fat in HFD-fed mice. 

Visceral fat accumulation and dyslipidemia are the key characteristics of HFD-induced NAFLD [40,41]. Lipid synthesis in lipid metabolism is a complex transcriptional cascade. AMPK (AMP-activated protein kinase) serves as a critical regulator of lipid metabolism [42,43]. Upon activation, AMPK inhibits the expression of lipogenic genes by attenuating the biological activity of sterol regulatory element-binding protein 1c (SREBP-1c) [42]. This regulatory mechanism encompasses key enzymes involved in fatty acid synthesis, including fatty acid synthase (FAS) and acetyl-CoA carboxylase 1 (ACC1) [42]. Interestingly, both low molecular weight chitosan and selenium have been demonstrated to activate the lipid metabolism gene AMPK while simultaneously suppressing the expression of lipogenesis-related genes, thereby reducing lipid accumulation [44,45]. In our study, HFD-fed mice that underwent intervention with LCS-SeNPs exhibited a significant reduction in epididymal fat weight, subcutaneous fat weight, perirenal fat weight, and hepatic lipid accumulation compared with the HFD group. These observations suggested that LCS-SeNPs might have exerted anti-obesity effects by modulating the expression of genes associated with lipid synthesis. 

The intestinal barrier, as the largest and most important biological barrier against the external environment, plays a vital role in resisting gut microbiota, endotoxins, and various antigens in the intestine [26]. Prolonged consumption of an HFD resulted in intestinal barrier disruption, which exacerbated increased intestinal permeability, leading to elevated levels of endotoxins and various inflammatory factors in the blood [46]. Endotoxins and inflammatory cytokines infiltrate the liver via the portal vein, further triggering or exacerbating NAFLD [47]. A recent study indicated that a platelet membrane (PM) cloaks Se nanoparticles (SeNPs) delivery system with chitosan (CS) modifies and miR-148a-3p inhibitors encapsulated exhibited excellent targeted delivery capabilities in hyperlipidemic mice, significantly reducing levels of inflammatory factors associated with hyperlipidemia [48]. In our study, we found that intervention with LCS-SeNPs in HFD-fed mice significantly reduced serum levels of LPS, DAO, TNF-α, and IL-6, which was accompanied by marked histopathological improvement in the liver (Figure 2A). Furthermore, a decrease in the levels of the tight junction proteins *ZO-1* and *occludin* can compromise the integrity of the intestinal barrier, leading to increased intestinal permeability [49,50]. This allows more harmful substances to pass through the gut, subsequently triggering NAFLD [51]. Moreover, as an important secreted protein, *Muc2* protein plays a key role in protecting the intestinal barrier and maintaining intestinal homeostasis [52]. Meanwhile, *Agr2* is pivotal in the biosynthesis of the *Muc2* protein [53]. Former research demonstrated that the mRNA expression levels of *Muc2* and *Agr2* in the colonic tissues of obese mice induced by an HFD were significantly reduced [54]. In this experiment, we also assessed the expression of these genes, and the results indicated that, compared with the model group, HFD-fed mice subjected to the LCS-SeNP intervention exhibited significant upregulation of *ZO-1*, *occluding*, *Muc2,* and *Agr2* genes. These changes were visually evident in the significantly thicker colonic mucus layer compared with the model group (Figure 3D), and in the increased mucin production within goblet cells, as observed under TEM (Figure 3F). Moreover, low molecular weight chitosan (LCS) did not achieve the same improvements in intestinal barrier function or reduction in inflammatory factor levels as LCS-SeNPs (Figure 3D and Figure 4). These findings corroborate the physiological alleviation of NAFLD symptoms in HFD-fed mice treated with LCS-SeNPs.

The disruptions in intestinal microbial balance are associated with obesity and correlated metabolic disorders in human beings [55]. Previous studies have suggested that both chitosan and selenium can individually target the intestinal flora to improve HFD-induced NAFLD [56,57,58]. However, there is currently no research on chitosan-based selenium composites as a functional food targeting the microbiota to alleviate NAFLD. In our study, we found that LCS-SeNPs can significantly increase the abundance of *Akkermansia*, which is a well-known beneficial bacteria [59,60]. Previous studies suggested that *Akkermansia* had remarkable effects on promoting the differentiation of goblet cells and protecting intestinal barrier function [61,62]. In our study, after the LCS-SeNP intervention, the amount of mucin secreted by goblet cells significantly increased, and the thickness of the mucus layer of HFD mice was restored. Moreover, the increase in *Bifidobacterium* abundance had beneficial effects on improving human health, particularly in reducing fat accumulation and improving gut dysbiosis in NAFLD induced by an HFD [63]. Here, we found that the relative abundance of *Bifidobacterium* was significantly higher in the LCS-SeNP group compared with the HFD and LCS groups. This suggested that the improvement of NAFLD symptoms by LCS-SeNPs might have been due to the increased abundance of *Bifidobacterium*. Furthermore, earlier investigations had suggested a decline in the prevalence of *Muribaculaceae_unclassified* under HFD treatment [64,65]. Interestingly, our study confirmed that after LCS-SeNP treatment, there was a restoration in the abundance of *Muribaculaceae_unclassified*. We also observed a reduction in the abundance of several harmful bacteria to varying degrees in the group treated with LCS-SeNPs, such as the genus *Anaerotruncus*, *Lachnoclostridium*, and *Proteus.* As reported, there exists a direct association between the abundance of these detrimental bacteria and the incidence of obesity in mice [37,66,67].

Bile acids and their receptors are considered latent therapeutic targets for intervening in NAFLD [68]. The farnesoid X receptor (FXR) is a nuclear receptor that regulates bile acid metabolism [68,69]. Tauro-β-muricholic acid (T-β-MCA) acts as an antagonist to FXR and can disrupt lipid metabolism when excessively produced [68,69]. According to Spearman’s correlation analysis, our results showed a significant negative correlation between *Akkermansia* and T-β-MCA, suggesting that *Akkermansia* may alleviate NAFLD by reducing the fecal metabolite levels of T-β-MCA. In addition, microbes can deconjugate the primary bile acids and cholic acids from glycine or taurine conjugates via bile salt hydrolases, followed by related secondary bile acids that protect the intestinal epithelial barrier and inhibit obesity by increasing intestinal mucus [70,71,72,73]. Significant increases in glycocholic acid and taurodeoxycholic acid were observed under HFD treatment, indicating a strong association between these two conjugated bile acids and obesity [74,75]. Previous research indicated that *Bifidobacterium* contained various types of bile salt hydrolases, which endow it with a pivotal role in modulating the signaling pathways of bile acids and influencing lipid absorption [76]. Interestingly, our study results demonstrated that both glycocholic acid and taurodeoxycholic acid are negatively correlated with *Bifidobacterium* in the LCS-SeNP group, suggesting that LCS-SeNPs may regulate bile acid metabolism by increasing the abundance of *Bifidobacterium*. Additionally, *Muribaculaceae_unclassified* showed a significant positive correlation with gut homeostasis-maintaining metabolites such as kynurenic acid, indole, and 4-(2-aminophenyl)-2,4-dioxobutanoic acid. These results implied that LCS-SeNPs may potentially exert an inducing prebiotic effect in HFD mice. Glycerophospholipid metabolism exerts a fundamental influence in maintaining liver health and intestinal environmental homeostasis [77]. Lysophosphatidylethanolamines (LysoPE) serve as significant metabolites in glycerophospholipid metabolism, and LysoPE demonstrates the potential to alleviate disruptions in lipid and glucose metabolism in obese mice, and its lower content has been observed in individuals with NAFLD [78,79]. According to Spearman’s correlation analysis, harmful bacteria *Anaerotruncus*, *Lachnoclostridium*, and *Proteus* exhibited an obvious negative correlation with the metabolite LysoPE. This observation suggested that LCS-SeNPs may alleviate NAFLD by restoring glycerophospholipid metabolism levels through reducing the abundance of harmful bacteria. 

## 5. Conclusions

In conclusion, LCS-SeNPs significantly restore intestinal barrier damage induced by HFD and alleviate NAFLD. In contrast, LCS exhibits only partial efficacy in this regard. The therapeutic effects of LCS-SeNPs are attributable to its modulation of diverse metabolic pathways, which encompasses glycerophospholipid metabolism, primary bile acid metabolism, and tryptophan metabolism. This intervention significantly enhances the abundance of potentially advantageous bacteria within the intestinal tract, conducing to the restoration of gut homeostasis. It is noteworthy that the difference in the induced beneficial bacterial spectrum may be the reason why LCS-SeNPs have a stronger anti-obesity ability compared with LCS. Among these, *Akkermansia, Bifidobacterium,* and *Muribaculaceae_unclassified* may play crucial roles in ameliorating HFD-induced NAFLD. These bacteria may contribute to the production of conjugated bile acids and tryptophan derivatives. Overall, this study elucidates the complex mechanistic links between LCS-SeNPs and the composition and metabolism of gut microbiota, providing novel insights into the therapeutic management of NAFLD.

## Figures and Tables

**Table 1:** Significant metabolites associated with lipid metabolism (LCS-SeNPs vs. HFD).

Metabolites ^a^	Up/Down ^b^	Fold Change ^c^	VIP	*m*/*z*	RT	Metabolism Pathway
Retinyl ester **	Down	0.26	2.72	301.22	6.13	Vitamin digestion and absorption
2-Isopropylmalic acid *	Up	2.52	1.07	175.06	2.67	Valine, leucine, and isoleucine biosynthesis
L-Cysteinesulfinic acid *	Down	0.45	1.57	152.00	0.88	Taurine and hypotaurine metabolism
Cortisone **	Up	2.81	1.75	361.20	4.04	Steroid hormone biosynthesis
Corticosterone **	Up	2.80	2.20	347.22	3.03	Steroid hormone biosynthesis
Retinoyl beta-glucuronide **	Up	4.48	1.76	477.25	4.11	Retinol metabolism
Cytidine **	Down	0.34	2.31	244.09	1.32	Pyrimidine metabolism
7-alpha-hydroxy-4-cholesten-3-one **	Up	2.47	1.60	401.34	4.96	Primary bile acid biosynthesis
Glycocholic acid *	Down	0.30	1.97	464.31	5.92	Primary bile acid biosynthesis
Taurine **	Down	0.32	2.07	124.01	0.82	Primary bile acid biosynthesis
Taurodeoxycholic acid **	Down	0.28	2.01	498.29	3.42	-
Tauro-beta-muricholic acid *	Down	0.21	2.24	514.28	3.40	-
Glycerophosphocholine **	Down	0.47	1.58	258.11	1.33	Glycerophospholipid metabolism
LysoPE 14:0 *	Up	3.73	1.81	424.24	4.10	Glycerophospholipid metabolism
LysoPE 15:0 *	Up	4.72	2.05	438.26	4.32	Glycerophospholipid metabolism
TG 43:8; TG (12:2/12:2/19:4) **	Up	4.28	2.31	743.50	7.01	Glycerolipid metabolism
Oleic acid **	Up	2.02	2.78	327.25	6.78	Fatty acid biosynthesis

^a^ indicated a significant difference between the HFD and LCS-SeNP groups (* *p* < 0.05, ** *p* < 0.01). ^b^ “Up” and “Down” indicate the upregulation and downregulation trends in the LCS-SeNP group compared with the HFD group. ^c^ Fold change represented the extent of change in the intensity of metabolites in the LCS-SeNP group compared with the same metabolites in the HFD group. A fold change > 1 indicated that the metabolite intensity was higher in the LCS-SeNP group than in the HFD group, while a fold change < 1 signified that the metabolite intensity was lower in the LCS-SeNP group compared with the HFD group. LCS-SeNPs, low molecular weight chitosan selenium nanoparticles; HFD, high-fat diet; VIP, variable importance in projection; *m*/*z*, Mass to charge ratio; RT, Retention Time; LysoPE 14:0 and LysoPE 15:0, lysophosphatidylethanolamine; TG 43:8 and TG (12:2/12:2/19:4), triacylglycerol.

## Data Availability

The data presented in this study are available on request from the corresponding author.

## Supplementary Materials

The following supporting information can be downloaded at: https://www.mdpi.com/article/10.3390/jfb15080236/s1, Table S1: PCR primer sequences; Table S2: PCR reaction system; Table S3: PCR primer sequences for DNA detection; Table S4: Top 25 dominant bacteria in HFD+S group; Table S5: Top 25 dominant bacteria in HFD+C group; Figure S1: Improvement in intestinal flora structure by LCS-SeNP treatment in HFD mice; Figure S2: Changes in 10 representative differential microflora in HFD mice treated with LCS-SeNPs; Figure S3: Identification and correction of differential metabolites following LCS-SeNP treatment in HFD mice; Figure S4. The differential metabolites between the LCS-SeNPs and LCS groups.
