# Supplementary material for: Chitosan-Stabilized Selenium Nanoparticles Alleviate High-Fat Diet-Induced Non-Alcoholic Fatty Liver Disease (NAFLD) by Modulating the Gut Barrier Function and Microbiota
Source: J Funct Biomater. 2024 Aug 22;15(8):236. doi: 10.3390/jfb15080236 (PMC11355332; doi:10.3390/jfb15080236)
Supplement: Supplementary file 1 [file jfb-15-00236-s001.zip › jfb-3153125-supplementary.pdf]

Supplementary Materials

Chitosan-Stabilized Selenium Nanoparticles Alleviate High-Fat Diet-Induced Non-Alcoholic Fatty Liver Disease (NAFLD) by Modulating the Gut Barrier Function and Microbiota

Yuhang Luo [1], Shujiang Peng [1], Jintao Cheng [2], Hongli Yang [1], Lin Lin [1], Guiling Yang [2], Yuanxiang Jin [2,3], Qingchi Wang [2,*] and Zhengshun Wen [1,2,*]

[1] School of Food and Pharmacy, Zhejiang Ocean University, Zhoushan 316022, China
[2] Xianghu Laboratory, Hangzhou 311231, China
[3] College of Biotechnology and Bioengineering, Zhejiang University of Technology, Hangzhou 310032, China
* Correspondence: wangqingchi@xhlab.ac.cn (Q.W.); wenzhengshun@xhlab.ac.cn (Z.W.)

Table S1. PCR primer sequences.

Gene	Primer sequence（5' -3' ）
IL-6	F: TAGTCCTTCCTACCCCAATTTCC
	R: TTGGTCCTTAGCCACTCCTTC
TNF-*α*	F:CGTCAGCCGATTTGCTATCTCAT
	R:GGCCCACAGTCCAGGTCACT
ZO-1	F: TTTTTGACAGGGGGAGTGG
	R: TGCTGCAGAGGTCAAAGTTCAAG
Occludin	F: ATGTCCGGCCGATGCTCTC
	R: TTTGGCTGCTCTTGGGTCTGTAT
Agr2	F: GGCGATCAGCTCATCTGGACTCA
	R: AAGGCTTGACTGTGTGGGCATTC
Muc2	F:AGGGCTCGGAACTCCAGAAA
	R:CCAGGGAATCGGTAGACATCG
β-actin	F:GGCTGTATTCCCCTCCATCG
	R:CCAGTTGGTAACAATGCCATGT

Table S2. PCR reaction system.

Reagent	Volume（μL）
TB Green Premix Ex Taq (Tli RNaseH Plus)	10
PCR forward primer（10μM ）	0.4
PCR forward primer（10μM ）	0.4
ROX reference dye	0.4
DNA template	1
Sterile water	7.8
Total	20

Table S3. PCR primer sequences for DNA detection.

Amplified fragments	Primer sequence
V3 – V4	341F (5'-CCTACGGGNGGCWGCAG-3')
	805R(5'-GACTACHVGGGTATCTAATCC-3')

Table S4. Top 25 dominant bacteria in HFD+S group.

Genus	mean_HFD	mean_HFD+S	HFD+S/HFD
Akkermansia	1.23	32.75	26.59
Paramuribaculum	0.06	1.05	17.5
Bifidobacterium	0.02	0.18	9.20
Erysipelatoclostridium	0.44	1.78	4.02
Muribaculaceae_unclassified	7.10	16.37	2.31
Desulfovibrio	0.08	0.09	1.23
Colidextribacter	1.81	2.21	1.22
Lactobacillus	0.25	0.27	1.07
Firmicutes_unclassified	0.83	0.82	0.99
Anaerotignum	0.98	0.72	0.74
Muribaculum	0.07	0.05	0.69
Oscillibacter	0.98	0.55	0.56
Eisenbergiella	2.80	1.40	0.50
Tannerellaceae_unclassified	47.08	21.03	0.45
Ruminococcaceae_unclassified	1.49	0.60	0.40
Negativibacillus	1.66	0.63	0.38
Lachnospiraceae_NK4A136_group	3.64	1.24	0.34
Lachnospiraceae_unclassified	4.02	1.27	0.32
GCA-900066575	0.86	0.26	0.30
Clostridiales_unclassified	5.43	1.49	0.28
Lachnoclostridium	0.58	0.15	0.27
Roseburia	1.08	0.25	0.23
Anaerotruncus	5.34	1.11	0.21
Proteus	0.25	0.03	0.11
Acetatifactor	1.91	0.16	0.08

Table S5. Top 25 dominant bacteria in HFD+C group.

Genus	mean_HFD	mean_HFD+C	HFD+C/HFD
Desulfovibrio	0.08	3.6925	48.39
Akkermansia	1.23	7.7386	6.28
UCG-005	0.17	0.48	2.77
Eubacterium]_nodatum_group	0.18	0.4532	2.52
Erysipelatoclostridium	0.44	1.1158	2.52
Colidextribacter	1.81	2.1891	1.21
Ligilactobacillus	0.31	0.362	1.16
Tannerellaceae_unclassified	47.08	54.5724	1.16
Eisenbergiella	2.80	3.1628	1.13
Negativibacillus	1.66	1.6709	1.01
Romboutsia	0.39	0.326	0.83
Anaerotignum	0.98	0.8057	0.82
Parabacteroides	0.38	0.3106	0.82
Anaerotruncus	5.34	3.982	0.75
Muribaculaceae_unclassified	7.10	4.6566	0.66
Lachnoclostridium	0.58	0.3287	0.57
Ruminococcaceae_unclassified	1.49	0.8179	0.55
Roseburia	1.08	0.5747	0.53
Firmicutes_unclassified	0.83	0.4168	0.50
Oscillibacter	0.98	0.4821	0.49
Clostridiales_unclassified	5.43	2.6667	0.49
GCA-900066575	0.86	0.3592	0.42
Acetatifactor	1.39	0.5333	0.38
Lachnospiraceae_unclassified	4.02	1.4066	0.35
Lachnospiraceae_NK4A136_group	3.64	0.8955	0.25

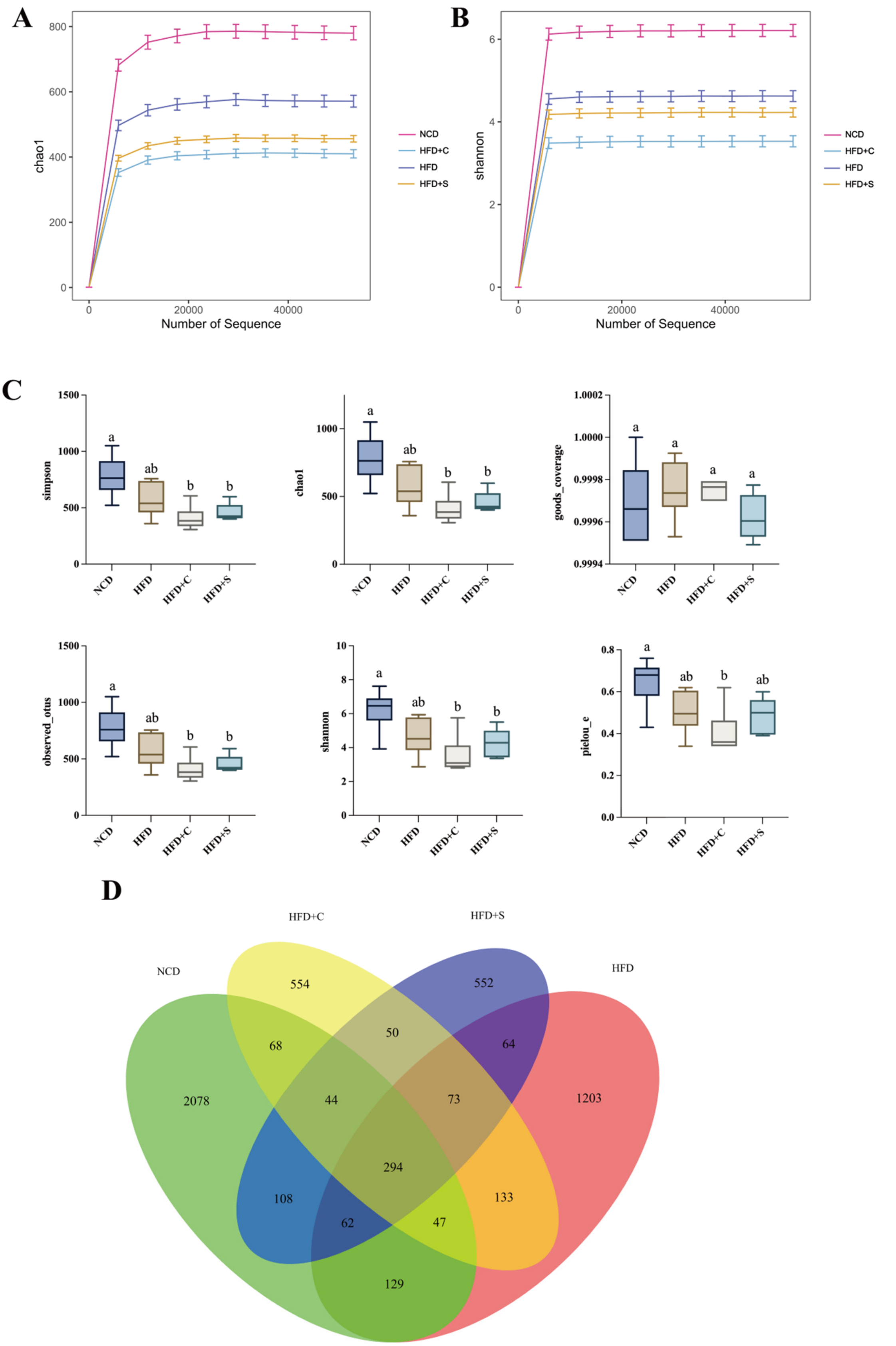

Figure S1. Improvement in intestinal flora structure by LCS-SeNPs treatment in HFD mice. (A) Chao1 rarefaction curves; (B) Shannon rarefaction curves; (C) 6 analyses of the Alpha diversity index; (D) ASV-Venn diagram. The distinct letters above the bar chart indicated significant differences ($p < 0.05$).

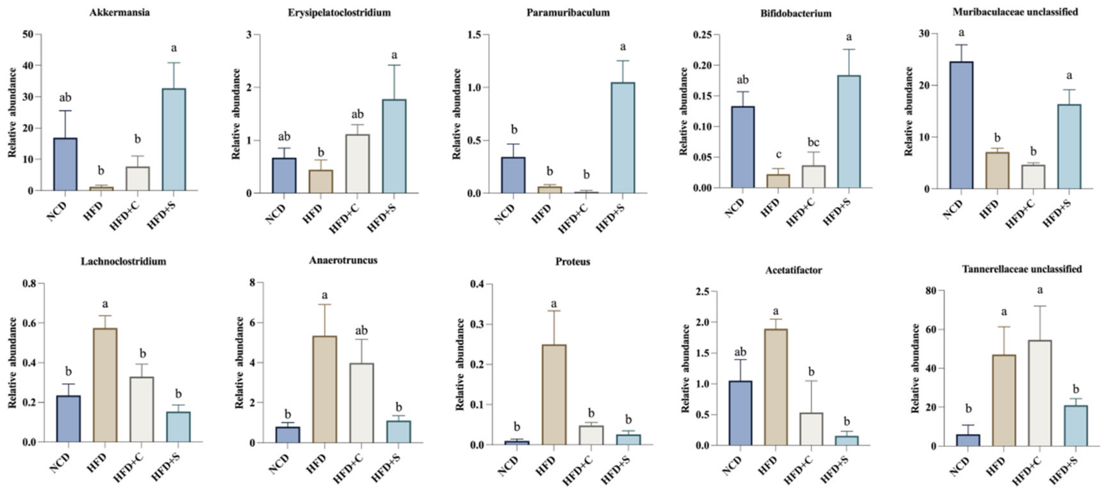

FigureS2. Changes of 10 representative differential microflora in HFD mice treated with LCS-SeNPs.

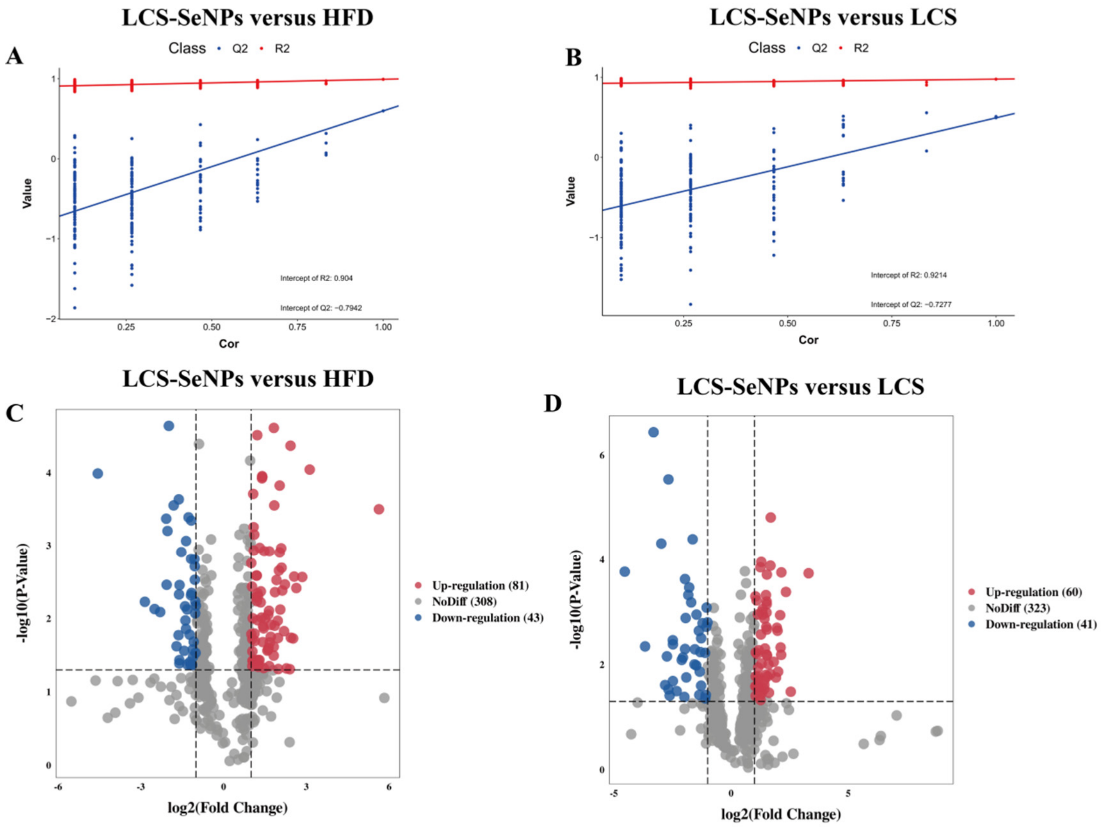

Figure S3. Identification and correction of differential metabolites following LCS-SeNPs treatment in HFD mice. Permutation test plots of PLSDA for the LCS-SeNPs versus HFD group with R2Y = 0.993 and Q2 = 0.598 (A), and the LCS-SeNPs versus LCS group with R2Y = 0.9768 and Q2 = 0.494 (B); Volcano map of metabolites identified by fecal metabolite, the LCS-SeNPs versus HFD (C) and the LCS-SeNPs versus LCS group (D).

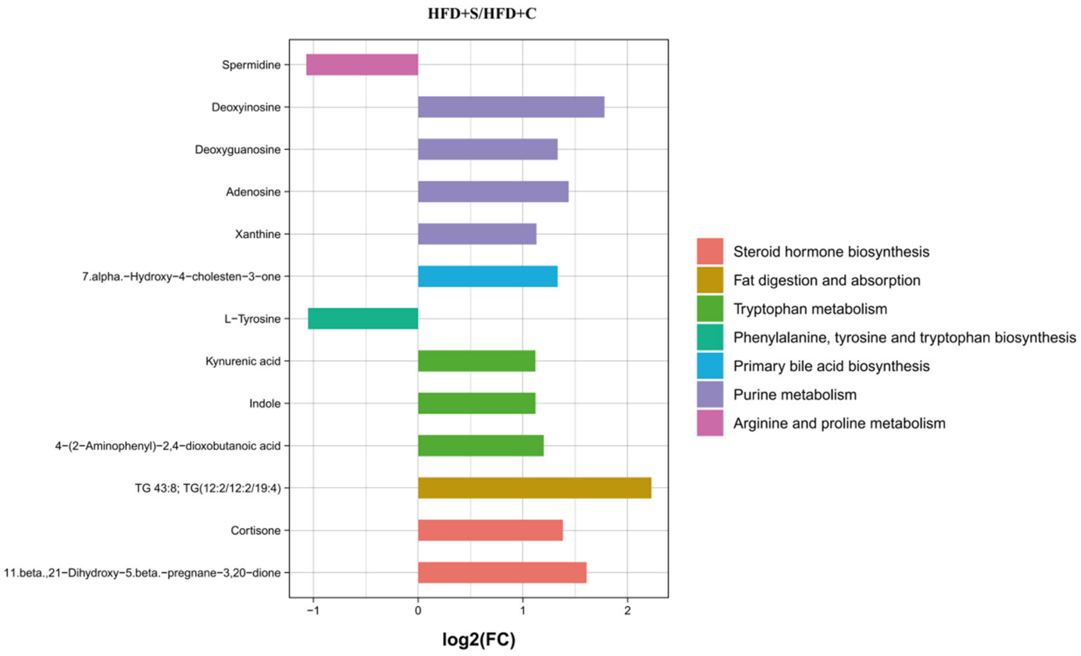

Figure S4. The differential metabolites between the LCS-SeNPs and LCS groups. Metabolites exhibiting notable distinctions between the LCS-SeNPs and LCS groups were depicted, with positive values on the X axis denoting upregulation and negative values indicating downregulation.
